# Deregulation of IL-37 and its miRNAs modulators in sarcopenic patients after rehabilitation

**DOI:** 10.1186/s12967-021-02830-5

**Published:** 2021-04-26

**Authors:** Francesca La Rosa, Simone Agostini, Marina Saresella, Andrea Saul Costa, Federica Piancone, Rossella Miglioli, Fabio Trecate, Mario Clerici

**Affiliations:** 1 IRCCS Fondazione Don Carlo Gnocchi-ONLUS, Milano, Italy; 2 Specialist Rehabilitation Unit, Istituto Palazzolo Don Carlo Gnocchi, Milano, Italy; 3 Department of Pathophysiology and Transplantation, University of Milano, Milano, Italy

**Keywords:** Cytokines, IL-37, Inflammaging, MiRNAs, Rehabilitation, Sarcopenia

## Abstract

**Background:**

sarcopenia is a highly prevalent condition in elderly individuals which is characterized by loss of muscle mass and functions; recent results showed that it is also associated with inflammation. Rehabilitation protocols for sarcopenia are designed to improve physical conditions, but very scarce data are available on their effects on inflammation We verified whether in sarcopenic patients the inflammation is reduced by rehabilitation and investigated the biological correlates of such effect.

**Methods:**

Twenty-one sarcopenic patients undergoing a specifically-designed rehabilitation program were enrolled in the study. Physical, cognitive and nutritional parameters, as well as the concentration of C-Reactive Protein (CRP), pro-and anti-inflammatory cytokines and cytokine production-modulating miRNAs were measured at the beginning (T_0_) and at end (30-days; T_1_) of the rehabilitation.

**Results:**

Rehabilitation resulted in a significant improvement of physical and cognitive conditions; this was accompanied by a significant reduction of CRP (p = 0.04) as well as of IL-18 (p = 0.008) and IL-37 (p = 0.009) concentration. Notably, the concentration of miR-335-3p (p = 0.007) and miR-657, the two known post-transcriptional regulators of IL-37 production, was increased by the rehabilitation protocol.

**Conclusions:**

Results herein confirm that successful rehabilitation for sarcopenia results in a reduction of the inflammatory milieu, raise the possibility that IL-37 may be a key target to monitor the rehabilitation-associated improvement in sarcopenia, and suggest that this cytokine could be a therapeutic target in sarcopenic patients.

## Background

Sarcopenia is a condition characterized by a progressive decline of muscle mass, quality, and strength [1, 2]. The prevalence of sarcopenia among people older than 65 years is estimated to be as high as 15%, and 50% of people over the age of 80 suffer from this condition [3, 4]. The European Working Group on Sarcopenia in Older People (EWGSOP) [5] developed definitions, diagnostic criteria, categories, and stages in sarcopenia and identified sedentary life as one of the main predisposing factors for age-related muscle loss [6–8]. Notably, sarcopenia is also associated with inflammation [9] within the so called inflammaging process: the preferential production of proinflammatory cytokines during aging, possibly as a consequence of an age-related redox imbalance that activates pro-inflammatory signaling pathways [10]. Inflammatory markers including interleukin 6 (IL-6), C Reactive Protein (CRP) and tumor necrosis factor-alpha (TNF-a) were shown to positively correlate with sarcopenia [11]. However, because several factors are known to affect the production of inflammatory proteins, it is difficult to understand their potential usefulness in the diagnosis and the monitoring of sarcopenia is difficult. More-in-depth analyses of vast panels of immune markers, thus, could help in the identification of new biomarkers for sarcopenia*.* Physical activity, such as aerobic exercise training, remains the most import strategy to prevent age-related muscle decline [12–16], but the observation that sarcopenia is associated with inflammation raised the possibility that interventions to reduce age-associate muscular degeneration could target such process [17, 18].

Inflammation is an extremely complex process that involves a number of different proteins. Interleukin 37 (IL-37), in particular, is a cytokine with anti-inflammatory properties that was shown to play a critical role in limiting excessive inflammatory responses [19, 20]. In humans, IL-37 production is not constitutive but is rather activated by pro-inflammatory stimuli, including the production of IL-1*β*, IL-18, TNF-*α*, IFN-*γ*, as a protective mechanism to prevent excessive inflammation and tissue damage [21]. As is the case with all genes, IL-37 expression is modulated by microRNAs (miRNAs), short non-coding RNAs (about 20–24 nucleotides) involved in mRNA silencing and post-transcriptional regulation via their ability to bind the 3′untranslated region (3′UTR). Data on miRNAs-mediated IL-37 regulation are scarce: very recent results obtained in women with a diagnosis of gestational diabetes mellitus showed that miR-657 modulates IL-37, influencing the inflammatory response [22]. In silico analysis (see mirtarbase and genecards) indicated that miR-335-3p can modulate the IL-37 expression as well.

In this pilot work we analyzed whether rehabilitation programs designed to improve function in sarcopenic patients would result in a reduction of inflammation. Results indicating that rehabilitation-associated improvement in the parameters used to monitor sarcopenia correlated with a significant reduction of IL-37 led us to investigate the post-transcription miRNA-mediated mechanism involved in IL-37 production, including miR-657 and miR-335-3p.

## Methods

### Participant recruitment

The study was conducted between January 2019 and September 2019 at the Palazzolo Institute, Don Gnocchi Foundation, Milan, Italy. Twenty-one patients were recruited (13 Females and 8 Males). Patients were diagnosed as being affected by severe sarcopenia according to the European Working Group on Sarcopenia in Older People (EWGSOP) [5]. All patients were hospitalized for 30 days; during this period they underwent the following rehabilitative treatment: twice daily session of 40′ in the morning and 30′ in the afternoon with assisted mobilization, progressive muscle strengthening, associated with progressivity of the load, standing work proprioceptive postural balance, walking training firstly with an assisted way and then without.

For each subject, Short Physical Performance Battery (SPPB) [23], Barthel Index [24, 25], and the Tinetti Balance Test to predict falls among individuals [26–28] were calculated before and after treatment; anthropometric measurements including age, sex, weight (kg) and height (cm) were measured while patients were wearing light clothes without shoes. The study conformed to the ethical principles of the Declaration of Helsinki; all subjects gave informed and written consent according to a protocol approved by the local ethics committee of the Don Carlo Gnocchi Foundation–ONLUS, Milan, Italy.

### Comprehensive geriatric assessment, nutrition status function and social behavior

At baseline all the individuals enrolled in the study underwent a comprehensive geriatric multidimensional evaluation that included cognitive function evaluation with Mini-Mental State Examination (MMSE) [29] and Clock Drawing test (CDT) (score range 0–5) [30]. Emotional status was evaluated via the Yesavage Geriatric Depression Scale (Yesavage GDS) [31] and comorbidity was evaluated with Charlson Comorbidity Index (CCI) [32]. Functional status was assessed using A.D.L. (activitiy of daily living or KATZ index) (bathing, dressing, toileting, transfer, feeding, continence) and I.A.D.L (Lawton-Brody instrumental activity of daily living) (telephone use, housekeeping, laundry, medication use, transportation, preparing meal, shopping, handling finances) tests [33–36]. Finally, Mini-Nutritional Assessment (MNA) was applied to all participants to analyze the nutritional status [37].

### Whole blood and plasma sampling and processing

About thirty milliliters of whole blood were collected in EDTA-containing vacutainer tubes (Becton Dickinson and Co., Rutherford, NJ, USA). Peripheral blood mononuclear cells (PBMCs) were separated on lymphocyte separation medium (Organon Teknika Corp., Durham, NC, USA), and washed twice in PBS. Leukocytes viability was determined using a Bio-Rad TC20 Automated Cell Counter (Bio-Rad, Hercules, CA, USA) and cryopreserved at − 80 °C in RPMI 1640 containing 50% fetal bovine serum (FBS) and 10% dimethylsulfoxide (DMSO) until using.

Plasma was obtained from blood collected in EDTA-containing vacutainer tubes (Becton Dickinson and Co., Rutherford, NJ, USA). Samples were prepared by one centrifugation at 2000*g* for 10 min (no brake) and stored at − 80 °C until testing.

### CRP-measurement

Plasmatic CRP was measured by turbidimetric method (lower detectable value: 0.1 mg/dL) by the DxC800 Chemistry Analyzer (Beckman Coulter, Brea, CA, US).

### ELISA

TNF-a, IL-6, IL-10, IL-18 and IL-37 concentration was determined in plasma by ELISA according to the manufacturer’s recommendations (Quantikine Immunoassay; R&D Systems, Minnepolis, MN, USA). The wells were read on a plate reader (Sunrise, Tecan, Mannedorf, Switzerland) and optical density (OD) was determined at 450/620 nm. The measured absorbance is proportional to the concentration of cytokines present in the plasma expressed in picogram per milliliter and calculated by dividing OD measurement generated from the assay by OD cut-off calibrator. All the experiments were performed in duplicate.

### miRNAs extraction and reverse transcription

miRNAs were extracted from 10 × 10^6^ PBMCs using a column-based kit (miRNeasy Mini Kit, Qiagen GmbH, Hilden, Germany) according to the manufacturer’s protocol. RNA concentration was determined by a spectrophotometer (Nanoview plus™, GE Healthcare, Little Chalfont, UK). Purity was determined as the 260/280 nm OD ratio, with the expected values between 1.8 and 2.0. RNA was treated with TURBO DNA-free DNAse (Ambion Inc., Austin, TX, US). Two-hundred ng of the extracted-RNA was retrotranscribed into cDNA using the universal cDNA synthesis kit (miRCURY LNA Universal cDNA synthesis kit, Exiqon (Inc by Qiagen GmbH, Hilden, Germany), according to the manufacturer’s protocol.

### Droplet digital PCR

miRNA quantitation was performed by droplet digital PCR (ddPCR, QX200, Bio-Rad, Hercules, CA, USA). Briefly, 3 μl of diluted cDNA (1:100) were mixed with LNA™-specific primers (has-miR-335-5p: cat. no: YP02119293, and has-miR-657: cat. no: YP02108736, Qiagen, GmbH, Hilden, Germany), and with ddPCR EvaGreen Supermix (Bio-Rad), which was then emulsified with droplet generator oil (Bio-Rad) using a QX200 droplet generator. The droplets were then transferred to a 96-well reaction plate and heat-sealed with a pierceable sealing foil sheet (PX1, PCR plate sealer, Bio-Rad). The PCR amplification was performed in sealed 96-well plate using a T100 thermal cycler (Bio-Rad) with the following cycling parameters: 10 min at 95 °C, 40 cycles at 94 °C for 30-s and at 58° for 60 s, followed by 10 min at 98 °C and a hold at 4 °C. After PCR amplification, the 96-well plate was transferred to a QX200 droplet reader (Bio-Rad). Each well was queried for fluorescence to determine the quantity of positive events (droplets), and the results were displayed as dot plots. The miRNA concentration was expressed as copies/ng of extracted RNA.

## Statistical analysis

Normally distributed data (clinical, demographic and cytokines) were expressed as mean ± standard deviation, and comparisons were analyzed by paired sample t-test. Not-normally distributed data (miRNA levels) were expressed as median and interquartile range (IQR), and comparison were analyzed by Wilcoxon signed-rank test (paired samples). Qualitative data were compared using Fisher's exact test, whereas the correlations were analyzed by Spearman's correlation coefficient.

Data analysis was performed using the MedCalc statistical package (MedCalc Software bvba, Mariakerke, Belgium). p-values of less than 0.05 were considered statistically significant.

## Results

### Effects of rehabilitation on physical and cognitive parameters

Epidemiological and clinical characteristics of the individuals enrolled in the study, as well as the effect of rehabilitation on these parameters are summarized in Table 1. Barthel Index (BI), SPPB score, and functional ability (Tinetti test) were evaluated in each patient at the beginning (T_0_) of the rehabilitation protocol and the end of rehabilitation, one month later (T_1_). Results showed that all these parameters were significantly improved by the rehabilitative treatment.

**Table 1.** Epidemiological and clinical characterization of the patients enrolled in the study

Sarcopenic patients	T_0_	T_1_	p-value
N°	21	21	
Gender (M:F)	8:13	–	
Age (years)	73.2 ± 8.9	–	
Education (years)	8.2 ± 3.13	–	
MMSE	28.8 ± 1.13	28.5 ± 2.27	*–*
CDT	3.40 ± 1.51	4.19 ± 0.84	*0.0026*
Yesavage GDS	8.14 ± 5.67	7.34 ± 5.85	*0.04*
CCI	3.61 ± 1.41	–	*–*
KATZ	2.88 ± 1.31	5.33 ± 0.67	*0.0001*
I.A.D.L (LAWTON-Brody instrumental activity)	2.89 ± 0.32	3.44 ± 1.05	*0,0084*

Data are expressed as mean ± standard deviation

Statistical analysis were performed using Wilcoxon test (paired samples); p-value (p) < 0.05 were considered statistically significant

MMSE: Mini-Mental State Examination; CDT: Clock Drawing test; GDS: Geriatric Depression Scale; CCI: Charlson Comorbidity Index

Elderly patients frequently suffer from complications related to comorbid condition, frailty and cognitive dysfunction. Results showed that these parameters were improved as well by rehabilitation. Thus, whereas no differences were seen in the MMSE score, all other scores were significantly improved at the end of the protocol (p < 0.005 in all cases) (Table 2). In particular, the clock drawing test (CDT) for executive cognitive functions, the Geriatric Depression Scale (GDS), and the Lawton Instrumental Activities of Daily Living (IADL) scale for evaluating the mood and general quality of life were all significantly increased at the end of rehabilitation. In contrast with these results, no differences were observed at the end of the rehabilitation in MNA, a parameter that analyzes the nutritional status (Table 3).

**Table 2.** Modification of mental and physical parameters induced by rehabilitation

Sarcopenic patients	T_0_	T_1_	p-value
N°	21	21	
Barthel	41.33 ± 21.59	78.59 ± 16.51	*0.0001*
SPPB-balance	0.29 ± 0.04	1.26 ± 0.7	*0.0003*
SPPB-walking	0.59 ± 0.41	1.29 ± 1.0	*0.0001*
SPPB-sit to stand	0.25 ± 0.65	1.03 ± 0.06	*0.0007*
Tinetti	10.96 ± 6.93	21.44 ± 3.20	*0.0001*

Data are expressed as mean ± standard deviation

Statistical analysis were performed using Wilcoxon test (paired samples); p-value (p) < 0.05 were considered statistically significant

MMSE: Mini Mental State Examination; SPPB: Short Physical Performance Battery; Tinetti: Balance test

**Table 3:** Nutritional status of the Sarcopenic patients enrolled in the study *Mini Nutritional Assessment (MNA)*

Sarcopenic patients	Range	> 24	> 17	17–23.5
T_0_	N°	3	9	9
	Means T_0_	29.8	18.76	11.6
T_1_	N°	3	10	8
	Means T_1_	32.9	19.4	12.5

The sum of the MNA score distinguishes between elderly patients:

(1) With adequate nutrition status (MNA > 24),

(2) With protein-calorie malnutrition (MNA > 17);

(3) At risk for malnutrition (MNA between 17 and 23.5).

With this scoring, sensitivity was found to be 96%, specificity 98%, and predictive value 97% [36]

### Effect of rehabilitation of inflammation as evaluated by C reactive protein

**Fig. 1:**
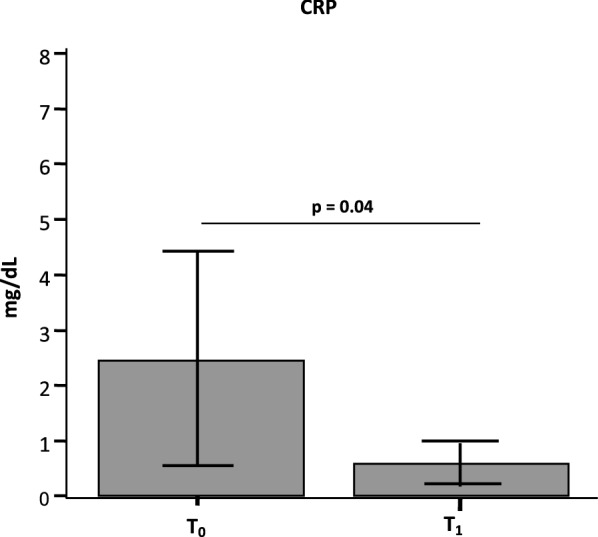
Plasma concentration of C-reactive (CRP) protein evaluated at baseline (T_0_) and at the end of the 30 days rehabilitation protocol (T_1_). Median CRP concentration (mg/dL) and statistical significance (paired samples t-test) are shown

Plasmatic concentration of CRP, an acute-phase protein of hepatic origin whose concentration raises in response to inflammation and is a common proxy to evaluate the presence of inflammatory conditions, was analyzed in all patients before and at the end of rehabilitation. Results showed that CRP plasma concentration was significantly reduced when results at T1 (0.9 mg/dL) were compared to those at baseline (3.03 mg/dL) (p = 0.04) (Fig. 1).

### Effects of rehabilitation on cytokines concentration

**Fig. 2:**
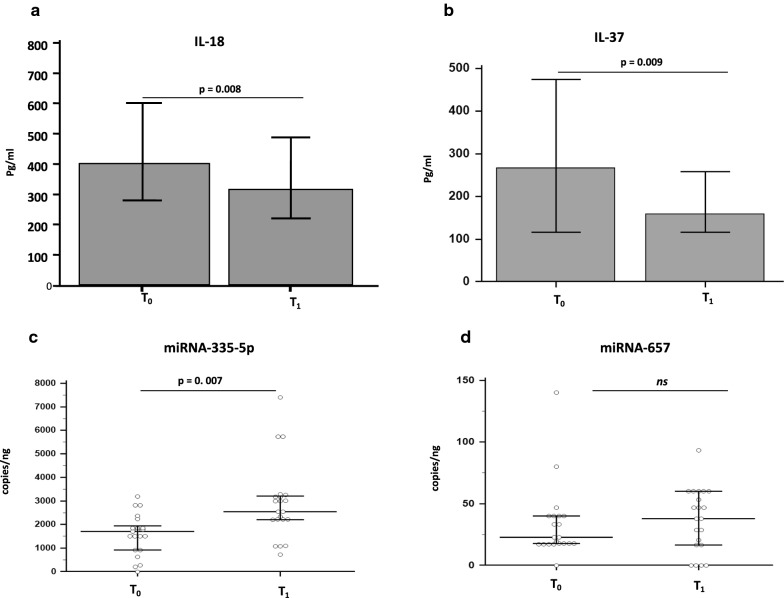
Interleukin-(IL)-18 (**a**) and Interleukin-(IL)-37 (**b**) plasma concentration at baseline (T_0_) and at the end of the 30 days rehabilitation protocol (T_1_). Median CRP concentration (mg/dL) and statistical significance (paired sample t-test) are shown

Cytokine production was evaluated by immunoassay (ELISA) in plasma of all the subjects enrolled in the study before (T0) and at the end (T1) of rehabilitation. Results showed that, whereas plasmatic concentration of TNF-α, IL-10 and IL-6 were comparable at the beginning and at the end of the study protocol (data not shown), IL-18 (p = 0.008) (Fig. 2a) and IL-37 (p = 0.009) (Fig. 2b) concentration was significantly reduced at T_1_ compared to T_0_.

### Effects of rehabilitation on miRNAs that modulate IL-37 production

**Fig. 3:**
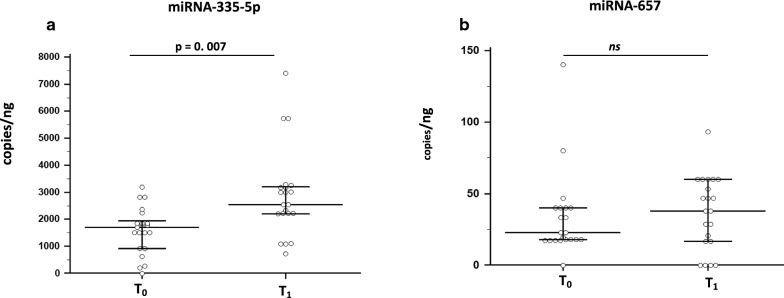
miRNA quantitation of miRNA-335-5p (**a**) and miRNA-657 (**b**) as performed by droplet digital PCR in RNA extracted by PBMC at baseline (T_0_) and at the end of the 30 days rehabilitation protocol (T_1_).Data are expressed as median and interquartile range (IQR). Statistical significance (Wilcoxon signed-rank test) is shown

Given that rehabilitation impacted on IL-37 concentration, we next examined the two miRNAs currently known to modulate the production of this cytokine. Thus, the expression levels of miR-335-5p and miR-657 was evaluated by ddPCR in cDNA extract from the same subjects enrolled in the study. A significant up-regulation of miR-335-5p concentration was observed at T_1_ (median: 2540 copies/ng) compared to the baseline values (T_0_) (median: 1700 copies/ng (p = 0.007) (Fig. 3a). miR-657 expression was upregulated as well at T_1_; the difference approached but did not reach statistical significance (median T_0_: 22.6 copies/ng; T_1_: 49.7 copies/ng) (Fig. 3b).

Finally, we verified whether there was a correlation between IL-37 and miR-335-5p expression in T0 and/or T1, but no correlation was found, probably due to the limited number of analyzed individuals.

## Discussion

The aging process is accompanied by a low-grade degree of inflammation in which increased levels of pro-inflammatory cytokines, CRP and a reduction of anti-inflammatory cytokines [38–40], as well as changes in immune responses [41] are seen. Aging is also associated with an increased prevalence of chronic conditions amongst which sarcopenia, a pathological alteration of muscle mass and functions, is highly common [42]. We investigated whether a multidimensional rehabilitation program designed for elderly sarcopenic patients could result in reduction of inflammation. Rehabilitation was successful, resulting in a significant improvement in physical and cognitive parameters; notably, rehabilitation also impacted on immunological parameters as it significantly reduced CRP as well as IL-18 and IL-37 plasmatic concentration, in parallel with an increase of miR-335-5p and miR-657 expression in PBMCs.

IL-18 is a pro-inflammatory cytokine released upon the activation of the NLRP3 multiprotein complex; such process leads to the downstream secretion of two important proinflammatory cytokines: IL-1β and IL-18 [43, 44]. IL-37, on the other hand, is one of the newest members of interleukin family and its concentration was shown to be increased in patients with different inflammatory and autoimmune diseases [20, 45, 46]. This cytokine was shown to be endowed with anti-inflammatory properties. Thus, available evidences [47–49] indicate that IL-37 serve as a natural brake of inflammation. To summarize: (1) IL-37 silencing via the use of IL-37-siRna in human blood monocytes results in an increased production of proinflammatory cytokines [19], and (2) transgenic mice expressing IL-37 (IL-37Tg) are protected against LPS challenge as a result of a reduced production of pro inflammatory cytokines and chemokines [19].

IL-37 production was shown to be induced by pro-inflammatory stimuli, including cytokines, as a protective mechanism to prevent excessive inflammation and tissue damage [19, 20]. Data herein show that IL-37 is augmented in elderly sarcopenic individuals, possibly in the attempt to generate a negative feedback to counteract aging-related systemic inflammation.

Notably, IL-18 and IL-37, the two cytokines modulated by rehabilitation, are functionally linked. Thus, IL-37 is the only member of the IL-1 cytokine family that binds to the IL-18 receptor (IL-18Rα). This prevents the interaction between IL-18Rα and IL-18, blocking the biological effects of IL-18. Il-37 can also recruit the orphan decoy IL-1R8, further reducing IL-18-dependent activation of innate and acquired immunity [50–53]. Other cytokines were evaluated in these patients, but rehabilitation did not affect their concentration; the IL-18/IL-37 functional interdependency might explain why these two cytokines alone were modulated. Efficient rehabilitation programs for sarcopenia, thus, could reduce inflammation and the need for IL-37 to exert its negative feedback to control the release of inflammatory cytokines.

Recent results indicated that at least two miRNAs, miR-657 and miR-335-5p, are involved in the post-transcriptional modulation of the activity of the IL-37 gene by targeting the IL-37 3′-UTR [22]. We investigated the possible effects of rehabilitation of these miRNAs and observed that both of them were increased at the end of the rehabilitation protocol, with the difference involving miR-335-5p being statistically significant. It should be underlined that the inverse association between IL-37 and it regulating miRNAs we detected at the end of the rehabilitation protocol is in line with the observation that the increased expression of a particular miRNA normally causes the decrease of the expression of the targeted mRNA and protein, in this case of IL-37. A limitation of the present work is the sample size: for this reason we are planning a case control study with larger samples size and control groups matched for age and comorbidities to overcome this limitation*.* Moreover, in the next future we will perform in vitro experiments to verify the interaction among IL-37, IL-18, miR-335-5p and miR-657, and in particular we are planning to analyze the relation between IL-37 and miR-335-5p, to verify the presence of possible specific interaction among these molecules and how they are involved in sarcopenia.

In conclusion results herein, although preliminary and needing to be confirmed in a bigger cohort, demonstrate that rehabilitation reduces inflammation and suggest that IL-37 and its regulatory miRNAs could be used as easily accessible biomarkers to evaluate the outcome of rehabilitation protocols for sarcopenia and, possibly, as therapeutic targets in this condition.

## Data Availability

The datasets generated and/or analyzed during the current study are available from the corresponding author on reasonable request.

## Abbreviations

ADL: Activitiy of daily living or KATZ index
BI: Barthel index
CCI: Charlson comorbidity index
CRP: C-reactive protein
CDT: Clock Drawing test
GDS: Geriatric depression scale
IADL: Lawton-Brody instrumental activity of daily living
IL-6: Intereukin-6
IL-18: Intereukin-18
IL-37: Intereukin-37
MMSE: Mini-mental state examination
SPPB: Short physical performance battery
